# Predictive biomarkers for post-neonatal necrotizing enterocolitis intestinal stenosis: Role of JMJD3, CRP, and PCT

**DOI:** 10.17305/bb.2025.12766

**Published:** 2025-08-13

**Authors:** Tao Qin, Chenxia Rao, Yunsen Deng

**Affiliations:** 1 Pediatrics Center, Wenling First People’s Hospital, Wenling, China; 2 Emergency Center, Zhongnan Hospital of Wuhan University, Wuhan, China; 3 Department of Neonatology, Shenzhen Bao’an Shiyan People’s Hospital, Shenzhen, China

**Keywords:** Intestinal stenosis, necrotizing enterocolitis, CRP, PCT, JMJD3

## Abstract

Necrotizing enterocolitis (NEC) is a severe, often life-threatening gastrointestinal disease in neonates, predominantly affecting preterm infants, and is frequently complicated by intestinal stenosis—a condition whose nonspecific clinical manifestations make early diagnosis and timely intervention particularly challenging. We aimed to investigate the clinical characteristics of NEC intestinal stenosis and its correlation with the histone demethylase Jumonji domain-containing protein 3 (JMJD3). A total of 310 children with NEC treated between February 2021 and June 2024 were retrospectively enrolled, categorizing them into an NEC group (*n* ═ 265) and a post-NEC intestinal stenosis group (*n* ═ 45). General data and laboratory indicators were collected, and analyses were performed to identify factors influencing the development of post-NEC intestinal stenosis. Spearman correlation analysis was utilized to assess relationships between JMJD3 and clinical parameters. Results indicated that the post-NEC intestinal stenosis group exhibited significantly lower platelet counts (PLT) and elevated levels of serum C-reactive protein (CRP), procalcitonin (PCT), and JMJD3 in intestinal tissues (*P* < 0.05). JMJD3 was significantly associated with the development of post-NEC intestinal stenosis (*P* < 0.001), presenting a 3.114-fold increased risk. Furthermore, JMJD3 levels were negatively correlated with PLT levels and positively correlated with CRP and PCT levels (*P* < 0.001). Receiver operating characteristic curve analysis demonstrated that the combination of CRP, PCT, and JMJD3 provided the highest predictive efficiency, with an area under the curve of 0.918, sensitivity of 86.67%, specificity of 86.42%, and a Youden index of 0.731 (*P* < 0.05), all surpassing the performance of individual markers. In conclusion, levels of CRP, PCT, and JMJD3 were significantly elevated in NEC children with intestinal stenosis. Their combined assessment presents a highly effective approach for the early diagnosis of post-NEC intestinal stenosis.

## Introduction

Necrotizing enterocolitis (NEC) is a severe intestinal disease primarily affecting neonates, particularly preterm infants, and is associated with infections of various etiologies. It is a common complication within this demographic and represents the most frequent gastrointestinal emergency encountered in the neonatal intensive care unit (NICU) [1, 2]. The incidence of NEC ranges from approximately 6% to 15%, with a mortality rate between 30% and 50%, thus posing a significant threat to neonatal morbidity and mortality [3, 4]. NEC can lead to intestinal necrosis or perforation, often resulting in various complications, with intestinal stenosis being one of the most prevalent [5]. The pathogenesis of post-NEC intestinal stenosis is not fully understood; however, it is thought to be linked to ischemia, necrosis, and injury to the intestinal wall secondary to NEC [6]. Early diagnosis of intestinal stenosis is challenging, often resulting in clinical misdiagnosis or delayed recognition, which can severely impact the growth, development, and quality of life of affected children. Despite advancements in early diagnosis and timely surgical interventions, such as enterectomy and anastomosis or enterostomy, the incidence of subsequent intestinal stenosis and obstruction remains notably high. Intestinal stenosis, which is the most common complication following NEC, has an incidence rate of 11%–35% [7]. In NEC patients managed conservatively with medical treatment, intestinal stenosis is predominantly observed in the transverse and descending colons. This may be attributed to the vulnerability of the terminal branches of the superior and inferior mesenteric arteries to ischemic injury. Conversely, in NEC patients who undergo surgical intervention, intestinal stenosis tends to occur in the ascending colon, likely due to the unique anatomical and functional characteristics of the ileocecal junction [8]. The non-specific clinical presentation of post-NEC intestinal stenosis often mimics feeding intolerance or gastroenteritis, complicating early diagnosis and increasing the risk of missed or delayed treatment, which adversely affects patient outcomes [9]. Therefore, identifying the risk factors for post-NEC intestinal stenosis and promoting early diagnosis and intervention are critical for improving prognosis in affected children.

Previous studies have demonstrated that intestinal inflammatory mechanisms are closely linked to complex molecular regulatory networks, including transcriptional modulation pathways [10]. In recent years, histone methylation modifications have garnered significant attention for their crucial roles in the pathogenesis and progression of various diseases, reflecting advancements in molecular biology. Jumonji domain-containing protein 3 (JMJD3), a histone demethylase, has been shown to be significantly upregulated in inflammatory conditions such as acute pancreatitis and autoimmune thyroiditis. JMJD3 enhances the expression of pro-inflammatory genes, thereby contributing to the development and exacerbation of inflammatory diseases [11, 12]. However, the role of JMJD3 as a contributing factor in the onset and progression of post-NEC remains unclear.

Given the severity of post-NEC intestinal stenosis and the potential involvement of JMJD3 in its pathophysiology, this study aims to investigate the clinical characteristics of post-NEC intestinal stenosis and their association with JMJD3. The goal is to offer new insights and strategies for the early diagnosis, targeted intervention, and prevention of post-NEC intestinal stenosis, ultimately enhancing patient prognosis and quality of life.

## Materials and methods

### Subjects

A total of 310 children diagnosed with NEC and treated at our hospital between February 2021 and June 2024 were retrospectively enrolled and categorized into either the NEC group or the post-NEC intestinal stenosis group, based on the presence or absence of intestinal stenosis.

The inclusion criteria were as follows: 1) children who met the diagnostic and treatment criteria for NEC, confirmed through clinical symptoms [13], imaging, and surgical pathology; 2) those classified in Bell stages I-III; 3) those who completed the diagnosis and treatment of NEC and post-NEC intestinal stenosis at our hospital; and 4) those with complete clinical case data. Exclusion criteria included: 1) children with congenital malformations of the heart, liver, kidneys, or gastrointestinal system; or 2) those with intestinal atresia, omphalocele, megacolon, or other gastrointestinal disorders. This study was approved by the Institutional Review Board of Wenling First People’s Hospital (No. ZJWL-F202112).

### Acquisition of general data

Data were collected on maternal factors such as gestational hypertension, gestational diabetes, mode of conception (natural conception or *in vitro* fertilization), and premature rupture of membranes. Additionally, neonatal factors were assessed, including mode of delivery (natural delivery or cesarean delivery), gender, gestational age, incidence of premature birth, birth weight, antibiotic use, hypoproteinemia, and history of blood transfusion.

### Detection of laboratory indicators

A total of 2 mL of venous blood was collected from each child and centrifuged using a high-speed centrifuge (Optima XPN, Beckman Coulter International Trading, Shanghai, China). The supernatant was then harvested for subsequent analyses. Serum white blood cell count (WBC), platelet count (PLT), and neutrophil ratio (NE) were measured with an automatic hematology analyzer (MEK-7222K, Shanghai Jumu Medical Instrument Co., Ltd., China). Serum albumin (ALB) levels were determined using an automatic biochemical analyzer (PUZS-300, Tips Biological, Shanghai, China) with a commercial kit obtained from Shanghai Ze Ye Biological Technology Co., Ltd. (catalogue number: ZY-ALB-Hu).

Serum levels of C-reactive protein (CRP) and procalcitonin (PCT) were quantified using enzyme-linked immunosorbent assay (ELISA) kits from Bioleaper, Shanghai, China (catalogue number: BR6000016), and Bio-Lab, Beijing, China (catalogue number: ARB13082), respectively.

For the detection of CRP levels, an appropriate volume of serum was added to each well of a microplate, followed by the addition of a primary antibody (anti-CRP antibody). The plate was incubated for 2 h at room temperature or overnight at 4 ^∘^C. After washing to remove unbound antibodies, a horseradish peroxidase (HRP)-labeled secondary antibody was added and incubated for 1 hour at room temperature. Subsequently, 3,3’,5,5’-tetramethylbenzidine (TMB) substrate reagent was added and incubated for 30 min at room temperature in the dark. The reaction was terminated by the addition of stop solution, and the optical density (OD) was measured at 450 nm.

For PCT detection, serum samples were added to separate microplate wells, followed by overnight incubation with a capture antibody (anti-PCT antibody) at 4^∘^C. After washing, an HRP-labeled detection antibody was added and incubated for 1 h at room temperature. TMB substrate reagent was then added and incubated for 30 min at room temperature in the dark, followed by the addition of stop solution. The OD value at 450 nm was measured using a fully automated biochemical analyzer or a microplate reader purchased from Molecular Devices (Shanghai), model: SpectraMax i3x. The concentrations of CRP and PCT were calculated based on standard curves.

Western blotting was conducted to evaluate the expression of JMJD3 in intestinal tissues. For children in the NEC group, intestinal samples were obtained either: 1) from surgically resected inflammatory necrotic bowel segments during laparotomy in severe NEC cases, or 2) via biopsy of affected intestinal segments in instances requiring endoscopic or operative intervention for diagnosis or management. NEC patients managed non-surgically (i.e., conservatively) did not have available intestinal samples for Western blotting analysis and were excluded from this molecular detection subanalysis to prevent tissue sampling bias.

For the post-NEC intestinal stenosis group, intestinal tissues were collected from the surgical resection margin during corrective surgery. To mitigate sampling bias, all tissues were collected intraoperatively following standardized procedures by trained pediatric surgeons. The median time from resection to freezing was consistently maintained within 30 min to ensure sample integrity. Moreover, identical anatomical segments were consistently sampled to ensure data uniformity. All tissue samples were immediately snap-frozen in liquid nitrogen after surgical excision and stored at −80 ^∘^C until protein extraction. Prior to analysis, tissues were randomly selected from available samples to ensure representation of anatomical locations and were handled by blinded personnel to reduce operator bias.

Total proteins were extracted from the intestinal tissues using lysis buffer (RIPA buffer, Shanghai Aladdin Biochemical Technology Co., Ltd., China, catalogue number: R493085), followed by high-speed centrifugation to collect the supernatant. The extracted proteins were then separated by SDS-PAGE (Wanleibio, China, catalogue number: WLA005a) and transferred to a polyvinylidene fluoride (PVDF) membrane. The membrane was blocked with non-specific antibodies (bovine serum ALB or skim milk) for 1 hour at room temperature to minimize non-specific binding. Subsequently, the blocked membrane was incubated overnight at 4 ^∘^C with a primary antibody against JMJD3 (Wuhan Booute Biotechnology Co., Ltd., China, catalogue number: orb1460584). The following day, the membrane was incubated with HRP-labeled secondary antibodies. Protein signals were visualized using an enhanced chemiluminescence (ECL) substrate (Bio-Rad Laboratories, catalogue numbers: 1705060, 1705061, and 1705062), and an X-ray film was utilized to record the signal intensity of the bands. Signal intensity is directly proportional to the expression level of the target proteins, with results frequently normalized to glyceraldehyde 3-phosphate dehydrogenase (GAPDH).

### Statistical analysis

Statistical analysis was conducted using SPSS 24.0 software. The normality of continuous variables was assessed with the Shapiro–Wilk test. Normally distributed data were presented as mean *±* standard deviation (*x ± s*) and compared between groups using the independent-samples *t*-test. Non-normally distributed data were expressed as median (interquartile range) and analyzed with the Mann–Whitney *U* test. Categorical data were reported as [*n* (%)] and analyzed using the chi-square (*χ^2^*) test.

To evaluate the correlation between JMJD3 expression and laboratory indicators, Spearman’s rank correlation coefficient (ρ) was employed for all variables, ensuring robust inference regardless of distribution type.

To identify independent risk factors for post-NEC intestinal stenosis, multivariate logistic regression analysis was performed. Candidate variables were selected based on clinical relevance and prior literature, including CRP, PCT, JMJD3, and other general or laboratory indicators with *P* < 0.10 in univariate analyses. Prior to inclusion, multicollinearity among predictors was evaluated using variance inflation factors (VIFs), with variables exhibiting VIF > 5 being excluded. A backward stepwise logistic regression approach was implemented, with an inclusion criterion of *P* < 0.05 and an exclusion criterion of *P* > 0.10. The logistic regression model was structured as Logit(*P*) ═ constant term + X1 × B1 + X2 × B2 + X3 × B3 +..., and the odds ratio (OR) along with the 95% confidence interval (CI) were calculated.

Receiver operating characteristic (ROC) curves were generated to evaluate the predictive performance of CRP, PCT, and JMJD3. A multivariate logistic regression model was developed to derive a logistic score for the combined prediction model of CRP, PCT, and JMJD3, which was subsequently analyzed using ROC curves. To assess the robustness and potential overfitting of the logistic regression model incorporating JMJD3, CRP, and PCT, internal validation was conducted through bootstrap resampling (1000 iterations). In each iteration, a bootstrap sample was drawn with replacement while preserving the original class proportions. The corresponding optimism-corrected area under the curve (AUC), sensitivity, and specificity were calculated across all bootstrap iterations and reported as mean values with 95% CIs. A *P* value of <0.05 was considered statistically significant.

## Results

### General data of NEC children with intestinal stenosis

All indicators of maternal and neonatal conditions demonstrated no statistically significant differences between NEC and post-NEC intestinal stenosis groups (*P* > 0.05) (Table 1).

**Table 1:** General data of NEC children with intestinal stenosis [*n* (%), (*x ± s*)]

**Group**	**NEC group (*n* ═ 265)**	**post-NEC intestinal stenosis group (*n* ═ 45)**	***t/χ^2^***	***P***
*Maternal condition*				
Gestational hypertension	44 (16.60)	8 (17.78)	0.038	0.845
Gestational diabetes	20 (7.55)	4 (8.89)	0.097	0.756
Mode of conception			0.034	0.854
Natural conception	233 (87.92)	40 (88.89)		
*In vitro* fertilization	32 (12.08)	5 (11.11)		
Premature rupture of membranes	24 (9.06)	3 (6.67)	0.276	0.599
*Neonatal condition*				
Mode of delivery			1.600	0.206
Natural delivery	115 (43.40)	15 (33.33)		
Cesarean delivery	150 (56.60)	30 (66.67)		
Gender			0.103	0.748
Male	154 (58.11)	25 (55.56)		
Female	111 (41.89)	20 (44.44)		
Gestational age (week)	36.74±3.79	37.01±4.49	0.430	0.668
Premature birth	142 (53.58)	27 (60.00)	0.638	0.424
Birth weight (g)	2714.36±610.58	2728.33±716.03	0.138	0.890
Antibiotic use	164 (61.89)	28 (62.22)	0.002	0.966
Hypoproteinemia	51 (19.25)	7 (15.56)	0.344	0.557
Blood transfusion	194 (73.21)	39 (86.67)	3.733	0.053

NEC: Necrotizing enterocolitis; post-NEC: Post-necrotizing enterocolitis.

### Laboratory indicators in NEC children with intestinal stenosis

**Figure 1.**
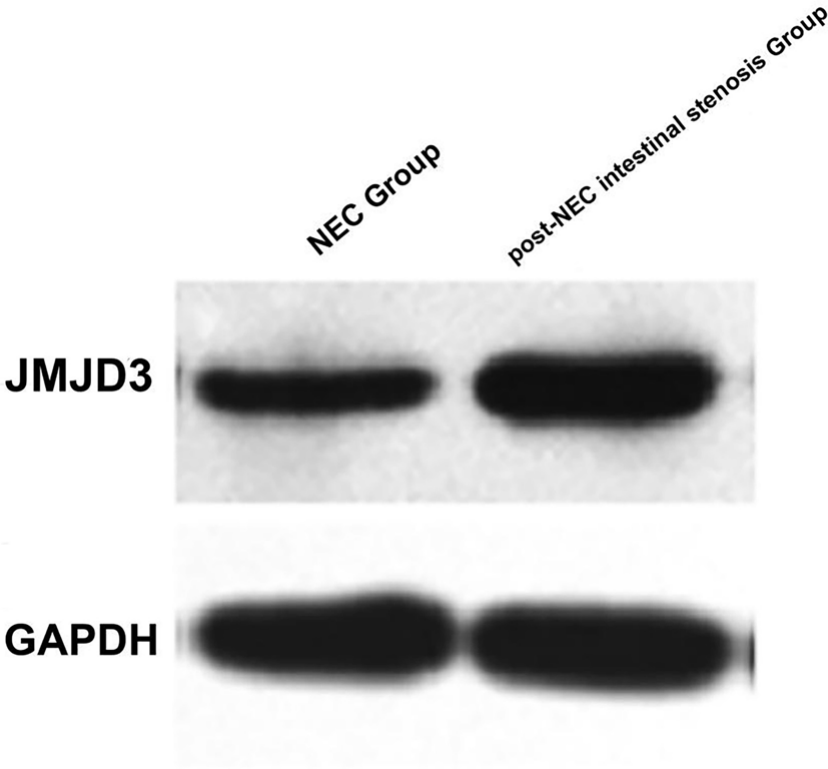
**Upregulation of JMJD3 protein expression in intestinal tissues of post-NEC intestinal stenosis patients compared with NEC group.** GAPDH was used as a loading control. JMJD3 expression was markedly elevated in the post-NEC intestinal stenosis group compared with the NEC group (*P* < 0.05). NEC: Necrotizing enterocolitis; GAPDH: Glyceraldehyde 3-phosphate dehydrogenase; JMJD3: Jumonji domain-containing protein 3.

Serum levels of WBC, ALB, and neutrophil elastase (NE) were comparable between the NEC group and the post-NEC intestinal stenosis group (*P* > 0.05). However, the post-NEC intestinal stenosis group demonstrated a significant decrease in PLT levels, alongside significant elevations in serum concentrations of CRP, PCT and JMJD3 expression in intestinal tissues when compared to the NEC group (*P* < 0.05) (Table 2). As illustrated in Figure 1, protein expression of JMJD3 was significantly upregulated in the intestinal tissues of the post-NEC intestinal stenosis group compared to the NEC group.

**Table 2:** Laboratory indicators in NEC children with intestinal stenosis (*x ± s*)

**Group**	**NEC group (*n* ═ 265)**	**post-NEC intestinal stenosis group (*n* ═ 45)**	***Z/t***	***P***
WBC (×10^9^/L)	12.10±3.20	12.84±3.74	1.398	0.163
PLT (×10^9^/L)	120.80±39.17	108.23±35.31	2.018	0.045
ALB (g/L)	31.74±2.45	32.13±3.71	0.907	0.365
NE (%)	0.67±0.17	0.65±0.15	0.742	0.459
CRP (mg/L)	48.5 (43.2, 54.8)	73.6 (61.7, 85.9)	6.124	<0.001
PCT (µg/L)	6.1 (5.2, 7.3)	13.2 (10.6, 15.9)	7.052	<0.001
JMJD3 expression	4.47±1.74	8.85±2.36	14.753	<0.001

WBC: White blood cell; PLT: Platelet count; ALB: Albumin; NE: Neutrophil ratio; CRP: C-reactive protein; PCT: Procalcitonin; JMJD3: Jumonji domain-containing protein 3.

### Multivariate analysis of factors associated with post-NEC intestinal stenosis

**Figure 2.**
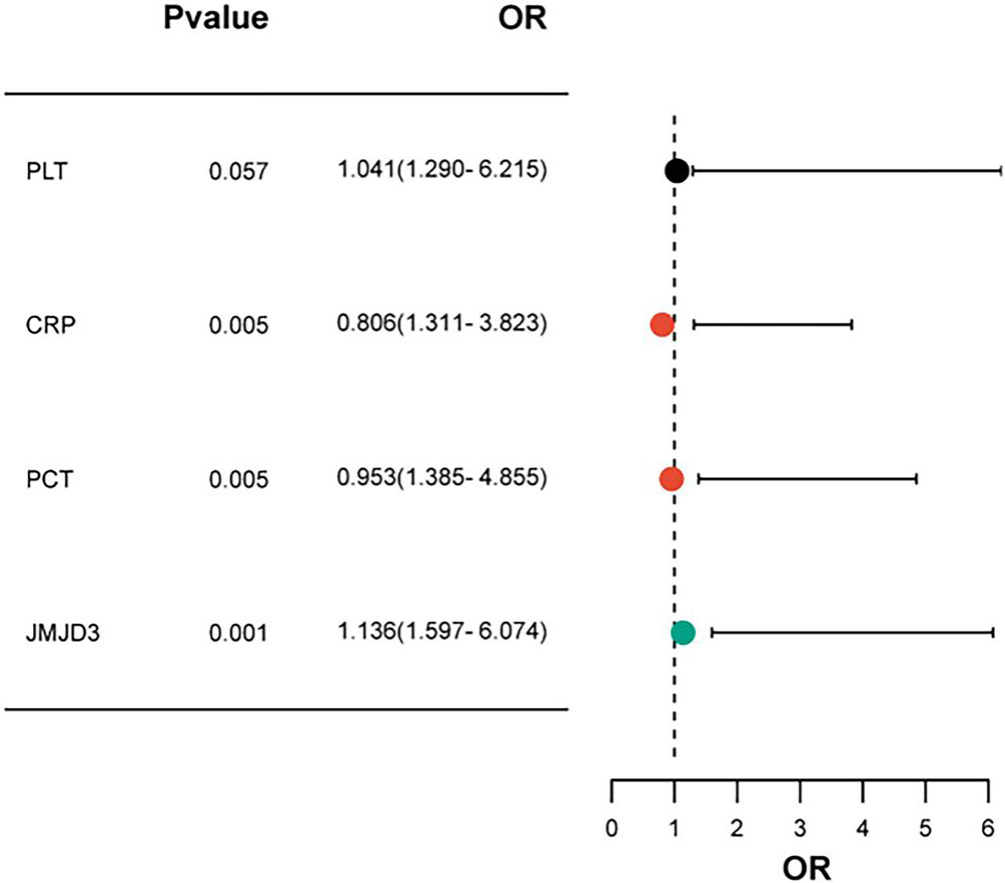
**Forest plot of multivariate analysis on the occurrence of post-NEC intestinal stenosis.** Red dots represent statistically significant predictors (*P* < 0.05), while grey dots denote non-significant predictors (*P* ≥ 0.05). post-NEC: Post-necrotizing enterocolitis.

Multivariate logistic regression analysis was performed to identify factors associated with the development of post-NEC intestinal stenosis. A backward stepwise logistic regression approach was utilized, and the complete list of candidate variables along with their elimination order is presented in Table S1. The analysis identified CRP, PCT, and JMJD3 as independent significant indicators (*P* < 0.05). Although PLT approached significance (*P* ═ 0.101), it did not meet the threshold for statistical significance. Elevated CRP levels (*P* ═ 0.005) and PCT levels (*P* ═ 0.005) were associated with increased risks of post-NEC intestinal stenosis, corresponding to 2.239-fold and 2.593-fold increases in risk, respectively. JMJD3 exhibited the strongest association (*P* < 0.001), with its upregulation linked to a 3.114-fold increase in the risk of developing intestinal stenosis (see Table 3 and Figure 2). CRP, PCT, and JMJD3 were retained as independent predictors of post-NEC intestinal stenosis, leading to the final logistic regression equation: Logit(P) ═ –5.321 + 0.806 × CRP + 0.953 × PCT + 1.136 × JMJD3, where *P* represents the predicted probability. Importantly, no significant multicollinearity was observed among the included variables (all VIFs < 2.0), which supports the reliability of the logistic regression model.

**Table 3:** Results of multivariate analysis on the occurrence of post-NEC intestinal stenosis

**Indicator**	**β**	**Standard error**	**Wald**	***P***	**Odds ratio**	**95% CI**
PLT	−1.041	0.635	2.690	0.101	0.353	0.098–1.272
CRP	0.806	0.273	8.717	0.005	2.239	1.311–3.823
PCT	0.953	0.320	8.869	0.005	2.593	1.385–4.855
JMJD3	1.136	0.341	11.098	<0.001	3.114	1.597–6.074

post-NEC: Post-necrotizing enterocolitis; CI: Confidence interval; PLT: Platelet count; CRP: C-reactive protein; PCT: Procalcitonin; JMJD3: Jumonji domain-containing protein 3.

### Results of correlation analysis

Spearman’s rank correlation analysis indicated a negative correlation between JMJD3 levels and PLT levels, alongside positive correlations with CRP and PCT levels (*P* < 0.001) (Table 4).

**Table 4:** Results of Spearman’s rank correlation analysis between laboratory indicators and JMJD3 expression

**Indicator**	**JMJD3**	
	***ρ***	***P***
PLT	−0.583	<0.001
CRP	0.476	<0.001
PCT	0.349	<0.001

PLT: Platelet count; CRP: C-reactive protein; PCT: Procalcitonin; JMJD3: Jumonji domain-containing protein 3.

### Efficiency of CRP, PCT, JMJD3 and their combination for predicting the development of post-NEC intestinal stenosis assessed by ROC curve analysis

**Figure 3.**
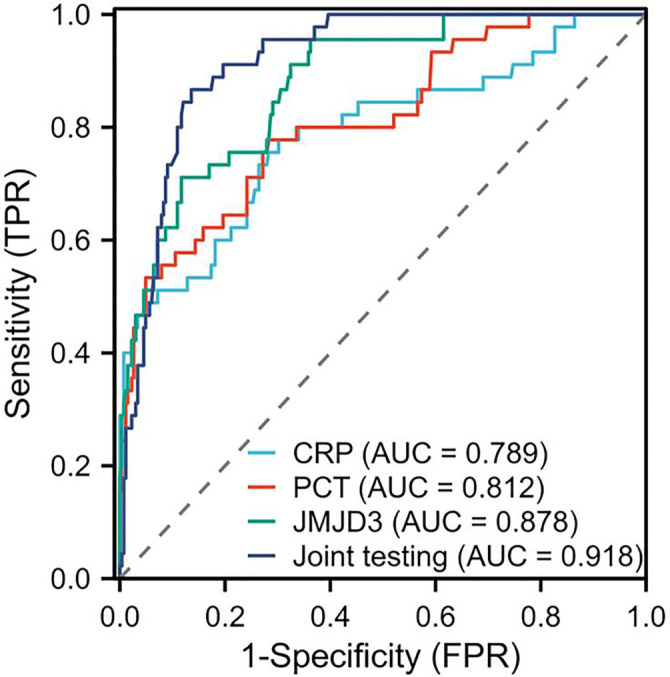
**ROC curve analysis on CRP, PCT, JMJD3 and their combination for predicting the occurrence of post-NEC intestinal stenosis.** ROC curves showing the predictive performance of CRP, PCT, JMJD3, and their combination for the diagnosis of post-neonatal NEC intestinal stenosis. Among individual biomarkers, JMJD3 achieved the highest AUC = 0.878, followed by PCT (AUC = 0.812) and CRP (AUC = 0.789). The combined logistic regression model incorporating CRP, PCT, and JMJD3 demonstrated the greatest predictive accuracy (AUC = 0.918), outperforming each single biomarker. ROC: Receiver operating characteristic; CRP: C-reactive protein; PCT: Procalcitonin; NEC: Necrotizing enterocolitis; AUC: Area under the curve; JMJD3: Jumonji domain-containing protein 3.

ROC curve analysis demonstrated that CRP had a relatively strong predictive value for post-NEC intestinal stenosis with an AUC of 0.789, (95% CI: 0.707–0.871), sensitivity of 77.78% (95% CI: 63.9%–87.7%), specificity of 69.81% (95% CI: 59.1%–78.7%), Youden index of 0.476, and a cut-off value of 56.28 mg/L (*P* < 0.05). PCT also exhibited good predictive performance, with an AUC of 0.812, (95% CI: 0.739–0.884), sensitivity of 77.78% (95% CI: 63.9%–87.7%), specificity of 71.70% (95% CI: 61.1%–80.5%), Youden index of 0.495, and a cut-off value of 9.35 µg/L (*P* < 0.05). JMJD3 showed the highest predictive accuracy among the individual biomarkers, with an AUC of 0.878, (95% CI: 0.827–0.929), sensitivity of 71.11% (95% CI: 56.3%–82.6%), specificity of 88.30% (95% CI: 79.4%–94.1%), Youden index of 0.594, and a cut-off value of 637.14 pg/mL (*P* < 0.05). Notably, the combination of CRP, PCT, and JMJD3 based on the logistic regression model score showed the highest predictive performance (AUC ═ 0.918, 95% CI: 0.884–0.952), with a sensitivity of 86.67% (95% CI: 73.8%–94.2%), specificity of 86.42% (95% CI: 77.3%–92.7%) and Youden index of 0.731 (*P* < 0.05), outperforming each single biomarker (Table 5 and Figure 3). The logistic regression model combining JMJD3, CRP, and PCT achieved a strong discriminative performance after internal validation. The optimism-corrected AUC based on 1000 bootstrap iterations was 0.892 (95% CI: 0.845–0.935). When applying the optimal cutoff derived from the Youden index in each bootstrap sample, the corrected sensitivity was 86.8% (95% CI: 68.9%–97.8%) and specificity was 81.1% (95% CI: 69.1%–93.2%). These findings support the high diagnostic utility of the model for predicting post-NEC intestinal stenosis.

**Table 5:** Efficiency of CRP, PCT, JMJD3 and their combination for predicting the occurrence of post-NEC intestinal stenosis assessed by ROC curve analysis

**Indicator**	**AUC (95% CI)**	**Sensitivity (95% CI)**	**Specificity (95% CI)**	**Youden index**	**Cut-off value**	***P***
CRP	0.789 (0.707–0.871)	77.78% (63.9–87.7%)	69.81% (59.1–78.7%)	0.476	56.28 mg/L	<0.05
PCT	0.812 (0.739–0.884)	77.78% (63.9–87.7%)	71.70% (61.1–80.5%)	0.495	9.35 µg/L	<0.05
JMJD3	0.878 (0.827–0.929)	71.11% (56.3–82.6%)	88.30% (79.4–94.1%)	0.594	637.14 pg/mL	<0.05
Combination	0.918 (0.884–0.952)	86.67% (73.8–94.2%)	86.42% (77.3–92.7%)	0.731	Predicted probability ≥0.63	<0.05

Although the combination model achieved the highest AUC, the 95% confidence intervals of the individual markers, particularly JMJD3, overlap, indicating that the performance gain may be incremental rather than statistically significant. CRP: C-reactive protein; PCT: Procalcitonin; ROC: Receiver operating characteristic; post-NEC: Post-necrotizing enterocolitis; JMJD3: Jumonji domain-containing protein 3; AUC: Area under the curve; CI: Confidence interval.

## Discussion

NEC is a prevalent acute abdominal condition in neonates, with most infants stabilizing after receiving active treatment during the acute phase. However, the incidence of intestinal stenosis among NEC patients is increasing annually [7]. Identifying the factors that contribute to the development of post-NEC intestinal stenosis, along with early prediction, diagnosis, and timely intervention, is essential for minimizing reoperation rates and enhancing long-term outcomes in affected children. This study explores the clinical and molecular characteristics of post-NEC intestinal stenosis and identifies potential predictive biomarkers. Our findings indicate that, while general perinatal parameters were similar between NEC patients with and without subsequent intestinal stenosis, significant differences were observed in specific inflammatory and molecular indicators. Notably, levels of CRP, PCT, and intestinal JMJD3 expression were markedly elevated in the stenosis group, while PLTs were reduced. These results suggest a systemic inflammatory response and epigenetic regulation may play critical roles in the development of post-NEC complications.

Low birth weight and premature birth are well-established risk factors for the development of post-NEC intestinal stenosis. In this study, we excluded these confounding variables to isolate additional influencing factors. Our analysis identified CRP, PCT, and JMJD3 as independent factors associated with post-NEC intestinal stenosis. Previous research has demonstrated elevated levels of inflammation-related factors in acute NEC, as well as persistent intestinal abnormalities, including stenosis, following disease resolution [14]. Intestinal stenosis, a common complication of inflammatory intestinal diseases, is believed to result from prolonged inflammatory responses and repetitive fibrotic remodeling of the intestinal wall [15].

CRP, an acute-phase protein, is significantly upregulated in response to inflammation, tissue injury, or infection through cytokine-mediated pathways. Elevated CRP levels correlate with more severe inflammatory responses [16]. The persistent elevation of CRP and abnormal lactate levels have been identified as predictors of post-NEC intestinal stenosis, which supports our findings [1]. Zani et al. [17] proposed that excessive inflammation, mediated by cytokine and chemokine release, disrupts intestinal mucosal integrity, impairs mucosal repair, and promotes the progression of NEC, ultimately leading to intestinal necrosis and perforation.

PCT, a precursor of calcitonin and a key marker of monocyte activation, rises early during inflammation and serves as a sensitive clinical biomarker [18]. In conjunction with intestinal oxygen saturation and mean platelet volume, serum PCT has demonstrated strong predictive value for assessing NEC severity [19]. Consistent with these findings, our study revealed that CRP and PCT expression levels were elevated in patients with post-NEC intestinal stenosis, with AUCs of 0.789 for CRP and 0.812 for PCT, indicating moderate to good predictive performance.

Previous studies have demonstrated that JMJD3 plays a critical role in inflammatory diseases. For example, JMJD3 expression is elevated in LPS-induced RAW264.7 cells, significantly enhancing the production of pro-inflammatory factors such as TNF-α, IL-1β, and IL-6 [20]. In osteoarthritis, increased JMJD3 expression activates the NF-κB signaling pathway, thereby promoting the inflammatory response [21]. Notably, JMJD3 has also been implicated in the regulation of intestinal inflammation, particularly during the progression of colitis. Research indicates that JMJD3 targets Nrf2 to modulate NLRP3 inflammasome activation, exacerbating colitis progression in mice treated with dextran sodium sulfate [22]. Furthermore, JMJD3 levels are elevated in patients with NEC and in neonatal mice subjected to experimental NEC, contributing to a pro-inflammatory response characterized by increased IL-6 and TNF-α release that drives NEC progression [23]. Collectively, these findings suggest that JMJD3 is closely involved in inflammatory intestinal diseases, including NEC. Our data reveal that JMJD3 expression is significantly increased in the intestinal tissues of patients with post-NEC intestinal stenosis, with ROC analysis yielding an AUC of 0.878.

The integration of CRP, PCT, and JMJD3 into a diagnostic panel yielded an enhanced AUC of 0.918. This combination surpassed the performance of any individual marker; however, the CIs for JMJD3 alone (AUC = 0.878; 95% CI: 0.827–0.929) and the combination model (AUC = 0.918; 95% CI: 0.884–0.952) exhibited substantial overlap, indicating that the improvement was incremental rather than statistically significant. Nonetheless, this combination significantly increased the Youden index from 0.594 to 0.731 and enhanced sensitivity from 71.11% to 86.67%. These improvements may offer considerable clinical advantages in real-world scenarios where heightened diagnostic accuracy is essential.

Given its central role in inflammatory processes, JMJD3 has garnered attention as a potential therapeutic target [24]. Mechanistically, JMJD3 acts as a histone H3K27 demethylase, which removes repressive epigenetic marks and enhances the transcription of pro-inflammatory genes [20]. One of the most studied pharmacologic inhibitors is GSK-J4, a cell-permeable prodrug that is converted intracellularly into the active JMJD3 inhibitor GSK-J1 [25]. GSK-J4 has demonstrated anti-inflammatory efficacy in various preclinical models, including colitis, arthritis, and sepsis, by downregulating IL-6, IL-17, and TNF-α, as well as attenuating inflammasome activation [26, 27]. These findings suggest that JMJD3 inhibition may alleviate intestinal inflammation. However, several challenges must be addressed before clinical translation, particularly in neonates [28–30].

This study has several limitations. First, while NEC patients were classified according to Bell stages I–III, no stratified analysis was conducted to evaluate JMJD3 expression or other relevant clinical indicators across different disease stages. Second, JMJD3 expression was measured at a single time point, and the absence of longitudinal evaluation limits our understanding of its temporal dynamics during disease progression and resolution. Future research should incorporate serial sampling at multiple stages of NEC and recovery to clarify the prognostic potential of JMJD3 in stricture development. Additionally, potential differences in the role of JMJD3 across diverse ethnic groups and clinical settings require further investigation to enhance broader clinical applicability. Continued research is expected to yield new breakthroughs and opportunities for the prevention and treatment of post-NEC intestinal stenosis.

## Conclusion

In conclusion, CRP, PCT and JMJD3 are significantly upregulated in NEC children with intestinal stenosis, and serve as key risk factors associated with the development of post-NEC intestinal stenosis. Moreover, the combined detection of these three biomarkers demonstrates high diagnostic efficiency for identifying affected individuals. Given the pivotal role of JMJD3 in the pathogenesis of post-NEC intestinal stenosis, it may work as a promising therapeutic target, thereby offering novel insights for future intervention strategies.

## Supplemental data

**Table S1:** Stepwise logistic regression process for predicting post-NEC intestinal stenosis

**Variable**	**Univariate *P* value**	**Stepwise status**	**β coefficient**	**Standard error**	**Wald**	***P***	**Odds ratio (95% CI)**	**Retained in final model**
Gestational hypertension	0.845	Not entered	–	–	–	–	–	–
Gestational diabetes	0.756	Not entered	–	–	–	–	–	–
Mode of conception	0.854	Not entered	–	–	–	–	–	–
Premature rupture of membranes	0.599	Not entered	–	–	–	–	–	–
Mode of delivery	0.206	Not entered	–	–	–	–	–	–
Gender	0.748	Not entered	–	–	–	–	–	–
Gestational age	0.668	Not entered	–	–	–	–	–	–
Premature birth	0.424	Not entered	–	–	–	–	–	–
Birth weight	0.890	Not entered	–	–	–	–	–	–
Antibiotic use	0.966	Not entered	–	–	–	–	–	–
Hypoproteinemia	0.557	Not entered	–	–	–	–	–	–
Blood transfusion	0.053	Eliminated in stepwise	–	–	–	–	–	No
WBC	0.163	Not entered	–	–	–	–	–	–
PLT	0.045	Trend retained	−1.041	0.635	2.690	0.101	0.353 (0.098–1.272)	No
ALB	0.365	Not entered	–	–	–	–	–	–
NE (%)	0.459	Not entered	–	–	–	–	–	–
CRP	<0.001	Retained	0.806	0.273	8.717	0.005	2.239 (1.311–3.823)	Yes
PCT	<0.001	Retained	0.953	0.320	8.869	0.005	2.593 (1.385–4.855)	Yes
JMJD3	<0.001	Retained	1.136	0.341	11.098	<0.001	3.114 (1.597–6.074)	Yes

JMJD3: Jumonji domain-containing protein 3; NEC: Necrotizing enterocolitis; WBC: White blood cells; PLT: Platelets; ALB: Albumin; NE (%): Neutrophil percentage; CRP: C-reactive protein; PCT: Procalcitonin; CI: Confidence interval.

## Data Availability

The data that support the findings of this study are available from the corresponding author upon reasonable request.
